# Association of institutional masking policies with healthcare-associated SARS-CoV-2 infections in Swiss acute care hospitals during the BA.4/5 wave (CH-SUR study): a retrospective observational study

**DOI:** 10.1186/s13756-024-01422-4

**Published:** 2024-06-18

**Authors:** Tamara Dörr, Sabine Güsewell, Domenica Flury, Maria Süveges, Camille Beatrice Gaza Valera, Sara Botero-Mesa, Marie-Céline Zanella, Anne Iten, Carlo Balmelli, Nicolas Troillet, Sarah Tschudin-Sutter, Peter W Schreiber, Philipp Jent, Lauro Damonti, Rami Sommerstein, Lea Portmann, Danielle Vuichard-Gysin, Alexia Cusini, Yvonne Nussbaumer-Ochsner, Ulrich Heininger, Christoph Berger, Petra Zimmermann, Céline Gardiol, Olivia Keiser, Matthias Schlegel, Philipp Kohler, Stefan P Kuster

**Affiliations:** 1 Division of Infectious Diseases, Infection Prevention and Travel Medicine, Cantonal Hospital St Gallen, Rorschacher Strasse 95, St. Gallen, CH-9007 Switzerland; 2 Faculté de Médecine de l’Université de Genève, Institut de Santé Globale, 24 rue du Général- Dufour, Genève 4, 1211 Switzerland; 3 Service de prévention et contrôle de l’infection, Direction médicale et qualité, Hôpitaux universitaires Genève, Rue Gabrielle-Perret-Gentil 4, Genève, 1205 Switzerland; 4 Infection Control Program and WHO Collaborating Centre on Patient Safety, Geneva University Hospitals and Faculty of Medicine, Rue Gabrielle-Perret-Gentil 4, Genève, 1205 Switzerland; 5 Infection Control Programme, EOC Hospitals, Viale Officina 3, Bellinzona, 6500 Switzerland; 6 Service of Infectious Diseases, Central Institute, Valais Hospitals, Av. Grand-Champsec 80, Sion, 1951 Switzerland; 7 Division of Infectious Diseases and Hospital Epidemiology, University Hospital Basel and University of Basel, Petersgraben 4, Basel, 4031 Switzerland; 8 Division of Infectious Diseases and Hospital Epidemiology, University Hospital Zurich and University of Zurich, Raemistrasse 100, Zurich, 8091 Switzerland; 9 Department of Infectious Diseases, Bern University Hospital (Inselspital), University of Bern, Länggassstrasse 122, Bern, 3012 Switzerland; 10 Faculty of Health Sciences and Medicine, Clinic St. Anna, University of Lucerne, Alpenquai 4, Lucerne, 6005 Switzerland; 11 Department of Infectious Diseases, Thurgau Cantonal Hospital, Spitalcampus 1, Muensterlingen, 8596 Switzerland; 12 Department of Infectious Diseases, Cantonal Hospital Graubuenden, Loëstrasse 170, Chur, 7000 Switzerland; 13 Klinik für Innere Medizin, Kantonsspital Spitäler Schaffhausen, Geissbergstrasse 81, Schaffhausen, 8208 Switzerland; 14 Infectious Diseases and Vaccinology, University of Basel Children’s Hospital, Spitalstrasse 33, Basel, 4056 Switzerland; 15 Division of Infectious Diseases and Children’s Research Centre, University Children’s Hospital Zurich, Steinwiesstrasse 75, Zurich, 8032 Switzerland; 16 Department of Community Health, Faculty of Science and Medicine, University of Fribourg, Chemin du Musée 8, Fribourg, 1700 Switzerland; 17 Department of Paediatrics, Fribourg Hospital HFR, Chemin des Pensionnats 2-6, Fribourg, Villars-sur-Glâne, 1752 Switzerland; 18 Swiss Federal Office of Public Health, Schwarzenburgstrasse 157, Bern, 3003 Switzerland

**Keywords:** Covid-19, Masking policies, Healthcare workers, Healthcare-associated infection, SARS-CoV-2

## Abstract

**Background:**

In the initial phase of the SARS-CoV-2 pandemic, masking has been widely accepted in healthcare institutions to mitigate the risk of healthcare-associated infection. Evidence, however, is still scant and the role of masks in preventing healthcare-associated SARS-CoV-2 acquisition remains unclear.We investigated the association of variation in institutional mask policies with healthcare-associated SARS-CoV-2 infections in acute care hospitals in Switzerland during the BA.4/5 2022 wave.

**Methods:**

SARS-CoV-2 infections in hospitalized patients between June 1 and September 5, 2022, were obtained from the “Hospital-based surveillance of COVID-19 in Switzerland”-database and classified as healthcare- or community-associated based on time of disease onset. Institutions provided information regarding institutional masking policies for healthcare workers and other prevention policies. The percentage of healthcare-associated SARS-CoV-2 infections was calculated per institution and per type of mask policy. The association of healthcare-associated SARS-CoV-2 infections with mask policies was tested using a negative binominal mixed-effect model.

**Results:**

We included 2’980 SARS-CoV-2 infections from 13 institutions, 444 (15%) were classified as healthcare-associated. Between June 20 and June 30, 2022, six (46%) institutions switched to a more stringent mask policy. The percentage of healthcare-associated infections subsequently declined in institutions with policy switch but not in the others. In particular, the switch from situative masking (standard precautions) to general masking of HCW in contact with patients was followed by a strong reduction of healthcare-associated infections (rate ratio 0.39, 95% CI 0.30–0.49). In contrast, when compared across hospitals, the percentage of health-care associated infections was not related to mask policies.

**Conclusions:**

Our findings suggest switching to a more stringent mask policy may be beneficial during increases of healthcare-associated SARS-CoV-2 infections at an institutional level.

**Supplementary Information:**

The online version contains supplementary material available at 10.1186/s13756-024-01422-4.

## Background

The wearing of a medical mask has been shown to be associated with a decreased risk of acquiring severe acute respiratory syndrome coronavirus type 2 (SARS-CoV-2) infection (1). In the living guideline on infection prevention and control of coronavirus disease (COVID-19), the World Health Organization (WHO) recommends all healthcare workers (HCW) to wear a well-fitting medical mask at all times within the health facility in areas of known or suspected community or cluster transmission (2). Inpatients are required to wear a medical mask if physical distancing of at least one metre cannot be maintained or when leaving their care area, provided there are no contraindications. The evidence base for this recommendation were five observational studies that found that implementing a universal masking policy in hospital systems was associated with a decreased risk of healthcare-acquired SARS-CoV-2 infection (3–7). It is acknowledged in the recommendation that these studies have limitations, mainly due to before-and-after design issues. Therefore, the certainty of evidence was considered “very low”. Nevertheless, as the literature provided only limited insight into associated detrimental effects, the WHO Guideline Development Group judged that the benefits of implementing universal mask use in healthcare facilities outweighed potential harms.

As a result of this recommendation, universal masking continues to be widely accepted in healthcare facilities worldwide, even with the emergence of the SARS-CoV-2 Omicron variant and widespread population immunity resulting from previous SARS-CoV-2 infection and/or vaccination (8). More recent evidence on this topic is currently unavailable, and expert opinion points towards using a mask for protection of all patients from all respiratory viral infections «when viral activity is elevated and for the most vulnerable patients year-round». However, these statements have been derived from mainly influenza-focused investigations (9).

In Switzerland, the mandatory indoor mask-wearing regulation was lifted on April 1, 2022, across all settings, including healthcare facilities. Thereafter, hospital policies on mask use were based on cantonal requirements or recommendations of Swissnoso, the Swiss National Center for Infection Control, that were regularly updated according to the epidemiological situation (10). Hence, masking policies varied between hospitals ever since, including the period of increased community circulation of BA.4/5 in summer 2022.

This study explores to what extent variation in institutional mask policy was associated with the occurrence of healthcare-associated SARS-CoV-2 infections in different institutions in Switzerland during the BA.4/5 2022 summer wave. We therefore analysed a national database that prospectively collected SARS-CoV-2 infections in hospitalized patients in large Swiss hospitals since 2020.

## Methods

### Patient-level data

Patient-level data was obtained from the COVID-19 Hospital Based Surveillance System (CH-SUR) database, which captured the details of COVID-19 cases from 20 large adult and pediatric hospitals in Switzerland. The study design and procedures of the CH-SUR study have been described previously (11). Data extraction and processing for this study are detailed in the supplement. All laboratory confirmed COVID-19 episodes (including polymerase chain reaction tests and antigen tests) with hospitalization for > 24 hours reported to the database were included. The mandatory information on date of hospital entry, demographics and episode declaration (classification, date of symptom onset, date of COVID-19 testing, laboratory result), as reported on the case report form, were used for our analysis.

Episodes were classified as either healthcare-associated (if symptom onset was > 5 days after admission date), or community-associated (earlier symptom onset). The time limit of 5 days was chosen to account for the incubation time of the virus, in line with the national recommendations of Swissnoso for healthcare-associated COVID-19 at the time of database development (12). The classification was provided by the reporting institution and verified using the reported dates of hospital entry and symptom onset. Episodes classified as “unknown” or "acquired from another healthcare institution" by the reporting institution were treated as community-associated in our verification and analysis.

### Institution-level data

All institutions participating in CH-SUR were invited to provide information about their infection prevention policies during the BA.4/5 wave, the time frame being set as 1 June to 31 August 2022, by means of a questionnaire. Questions included the total number of inpatient admissions and patient-days during this time period, mask policies (which could change over time), testing and screening policies and work policies for ill healthcare professionals; testing and screening policies as well as isolation precautions (including duration) for inpatients; and visitor restriction policies.

Mask policy – the main predictor variable – was classified as “standard precautions”, defined as wearing masks in contact with infected patients (policy 1); “mask in contact with patients”, defined as mandatory masking in contact with all patients regardless of diagnoses or symptoms (policy 2); “mask in contact with all contacts”, defined as mandatory masking in face-to-face contacts with all patients and also colleagues (policy 3) or “mask at all times”, indicating the policy where mask use was demanded regardless of activity or contact (policy 4). The term “mask” refers to at least a surgical mask. In most institutions, healthcare professionals could also opt for a respirator mask. If the mask policy changed during the time period considered, date(s) of switch were reported.

### Inclusion and exclusion criteria

For this study, we extracted all episodes in patients hospitalized between June 1 and September 5, 2022, and with a derived infection date (five days before symptom onset) between June 1 and August 31, 2022. Episodes with conflicting classification, i.e. classified as community-associated by the reporting institution but with documented disease onset > 5 days after admission or classified as healthcare-associated despite disease onset being documented within 5 days after admission, were excluded. Additionally, we excluded episodes whose incubation period could not be attributed to a mask policy, e.g. episodes with a derived infection date between June 1 and June 5, 2022, in institutions that changed the mask policy during this time.

### Outcome measures and statistical analysis

**Fig. 1:**
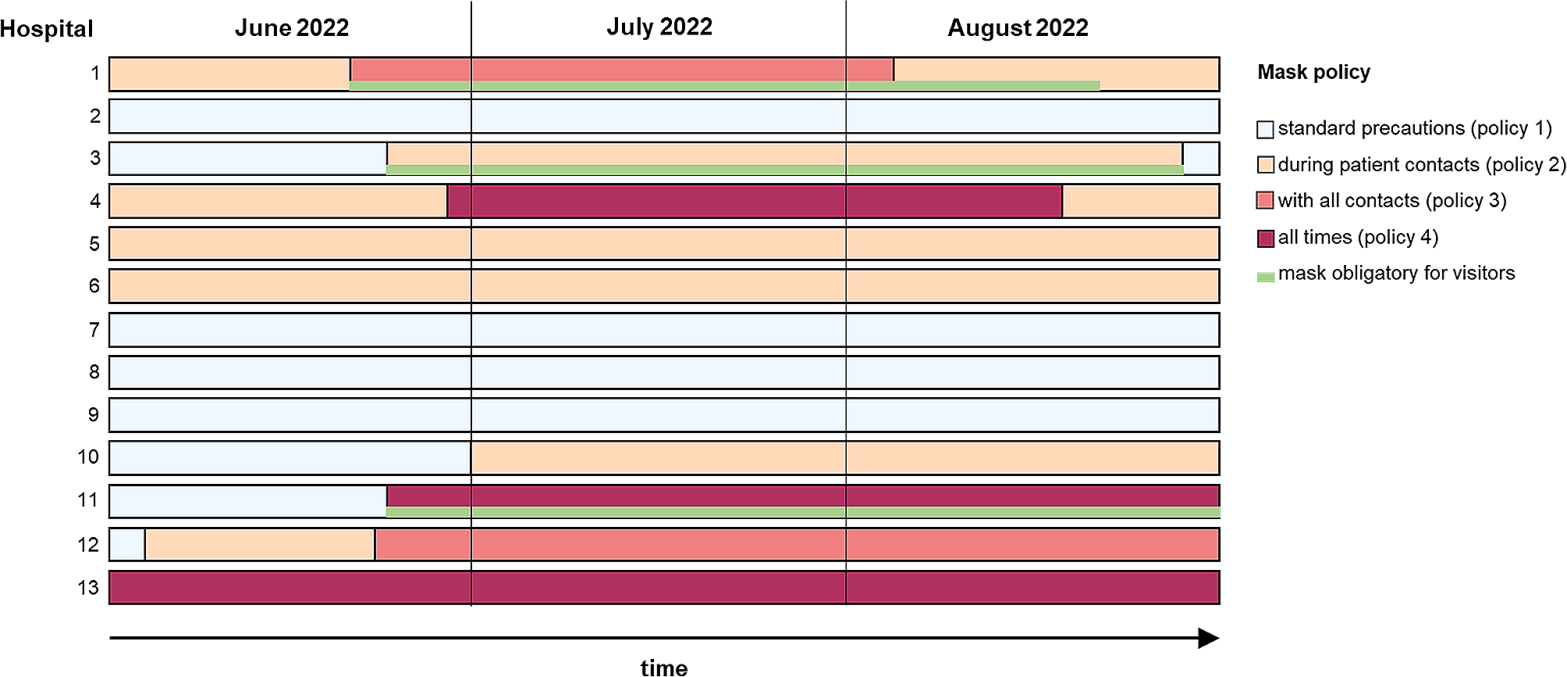
Time periods with different mask policies per institution between June 1 and August 31, 2022

Data were summarized by calculating the percentage of healthcare-associated SARS-CoV-2 infections per institution and (for institutions with policy switch) per mask policy, as the number of healthcare-associated cases divided by the total number of cases with SARS-CoV-2 infections in the same institution under the same mask policy. Attribution of SARS-CoV-2 episodes to a mask policy was based on derived infection date. If an institution switched back to its initial policy at the end of the time window (hospitals 1, 3 and 4, see Fig. 1), the two periods with identical mask policy were pooled. Percentages are shown with 95% Wilson confidence intervals.

The percentage of healthcare-associated SARS-CoV-2 infections was also calculated per institution for the entire time window to examine its association with the mean duration of stay per patient, a known risk factor for healthcare-associated SARS-CoV-2 (13). Because mean duration of stay (number of patient-days divided by number of admissions) was reported by institutions for the entire time window, this analysis only shows between-hospital differences.

The association of mask policy with the relative frequency of healthcare-associated SARS-CoV-2 infections (per institution and mask policy) was tested with a negative binominal mixed-effect model, including the number of healthcare-associated SARS-CoV-2 infections as outcome, the log-transformed number of community-acquired SARS-CoV-2 infections as offset (to standardize the outcome), mask policy as main predictor and mean duration of patient stay as covariable. To account for intra-hospital clustering, the institutions were included as random effects. In a subgroup analysis, the effect of switching from standard precautions to any policy including the masking of HCW in contact with patients was investigated. Only three hospitals performed this type of switch in late June 2022. Therefore, the model included hospitals as fixed effect to account for all between-hospital differences, and the two policy types as predictor.

Information about possible confounders (testing and screening policy, isolation precautions policy, visitor regulations, vaccination status) was not included in statistical analyses because, a) there was not much variation among institutions, and because b) the sample size did not allow more variables to be considered.

All analyses were performed in R, Version 4.02 (R Foundation, Vienna, Austria) using the packages lme4 and glmmTMB. Two-tailed tests were performed and *p*-values < 0.05 were considered statistically significant.

## Results

Fourteen hospitals participated in the study and provided institution-level data. Patient-level data were obtained from CH-SUR for these 14 hospitals. The dataset comprised 4’023 records which met the inclusion criteria; 115 reports were identified as duplicates belonging to the same episode, 316 were excluded due to symptom onset before June 6 (i.e. derived infection date outside the time window), 612 exclusions were made based on incomplete or inconsistent data. This also led to exclusion of one institution. Therefore, 13 institutions ended up being included in the analysis. Among the remaining 2’980 episodes, 444 (14.9%) were identified as healthcare-associated SARS-CoV-2 infection (see web-only Supplementary figure S1).

Six out of the 13 (46%) included institutions reported a switch to a more stringent policy between June 20 and June 30, 2022. Two institutions switched from policy 1 (standard precautions) to policy 2 (during patient contacts), two from policy 2 to policy 3, (with all contacts) and two switched from policy 1 or 2 to policy 4 (at all times). Three of these institutions made simultaneous changes to mask policy for visitors, requiring them to wear a mask when prior to that no restrictions were present. An overview of policies is given in Fig. 1. Other precautionary policies are detailed in the Supplement.

### Distribution of percentage of healthcare-associated infections between June 1 and August 31, 2022

**Fig. 2:**
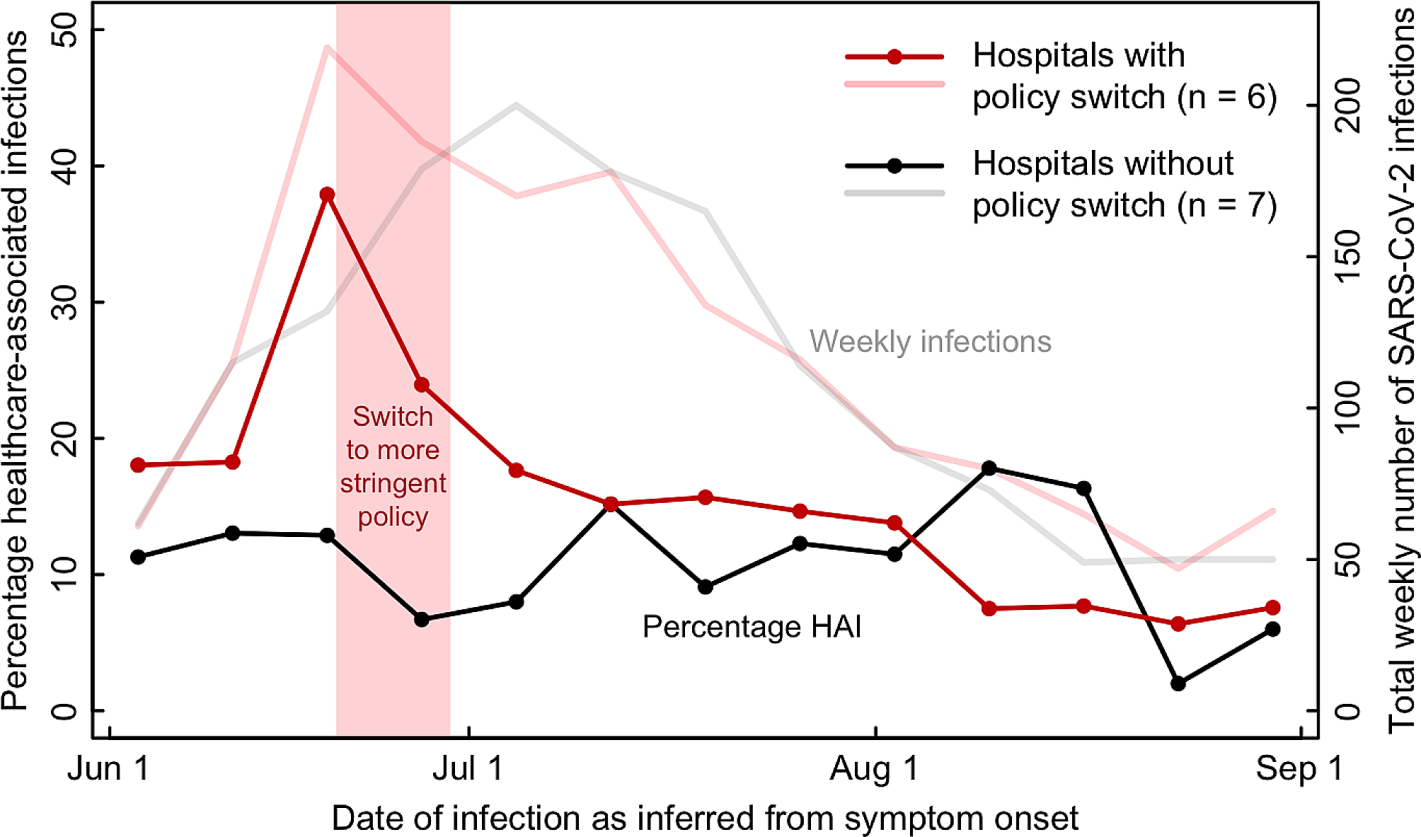
Weekly percentage of health-care associated infections (rich colors) and overall SARS-CoV-2 infection number per week (transparent colors), separately for institutions with and without mask policy switch. The red bar indicates the time period during which six institutions switched to a more stringent mask policy

The total number of SARS-CoV-2 infections increased until mid-June in the six institutions that switched to a more stringent mask policy, and until early July in the institutions without policy switch (Fig. 2). Subsequently the number of SARS-CoV-2 infections declined in both groups of institutions.

In institutions with policy switch, the percentage of healthcare-associated SARS-CoV-2 infections sharply increased before the switch, followed by a sharp and continuing decrease after the switch. Conversely, in institutions without policy switch, the percentage of healthcare-associated SARS-CoV-2 fluctuated with a slightly increasing trend until mid August (Fig. 2).

#### Percentage of healthcare-associated infections

**Fig. 3:**
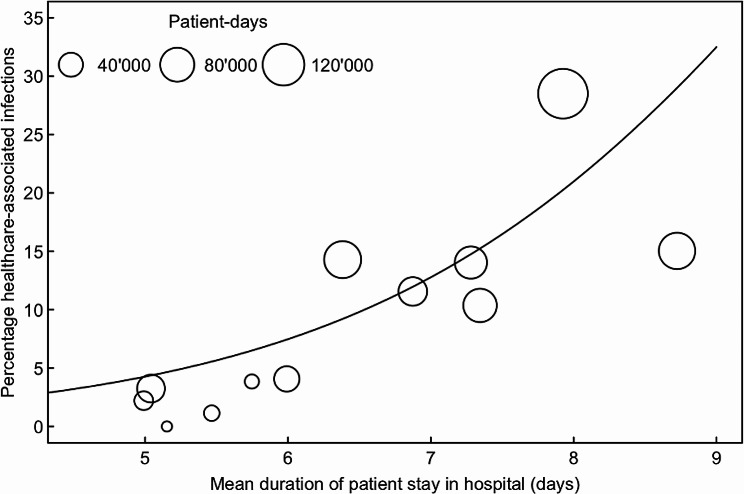
Percentage of healthcare-associated SARS-CoV-2 infections in relation to mean duration of patient stay. Each circle represents one hospital, circle size is proportional to hospital size (total number of patient-days from June to August 2022); hospital 11 is not included due to missing data on duration of patient stay

Across institutions, there was a wide variation in the percentage of healthcare-associated SARS-CoV-2 infections. This variation was related to the mean duration of patient stay: hospitals with longer stays reported higher percentages of healthcare-associated SARS-CoV-2 infections (Fig. 3). Furthermore, statistical modelling revealed a statistically significant association of healthcare-associated infection rates with mask policies after adjusting for mean duration of patient stay (Table 1): compared with standard precautions, all other mask policies were associated with a reduced rate of healthcare-associated infections (rate ratios; RR < 1).

**Table 1:** Negative binomial mixed-effects model evaluating the association of mask policies with the rate of healthcare-associated SARS-CoV-2 infections (number of healthcare-associated infections relative to number of community-acquired infections) both among and within institutions

*Effect in model*	RR (95% CI)	*p*-value
Mask with patients vs. standard	0.56 (0.32–0.96)	0.035
Mask during all contacts vs. standard	0.73 (0.21–2.53)	0.615
Mask indoors at all times vs. standard	0.40 (0.31–0.52)	< 0.001
Mean duration of patient stay (per day)	2.28 (1.58–3.27)	< 0.001
Overall test for differences among mask policies		< 0.01

RR: Rate ratio; *p*-values: Wald tests.

**Fig. 4:**
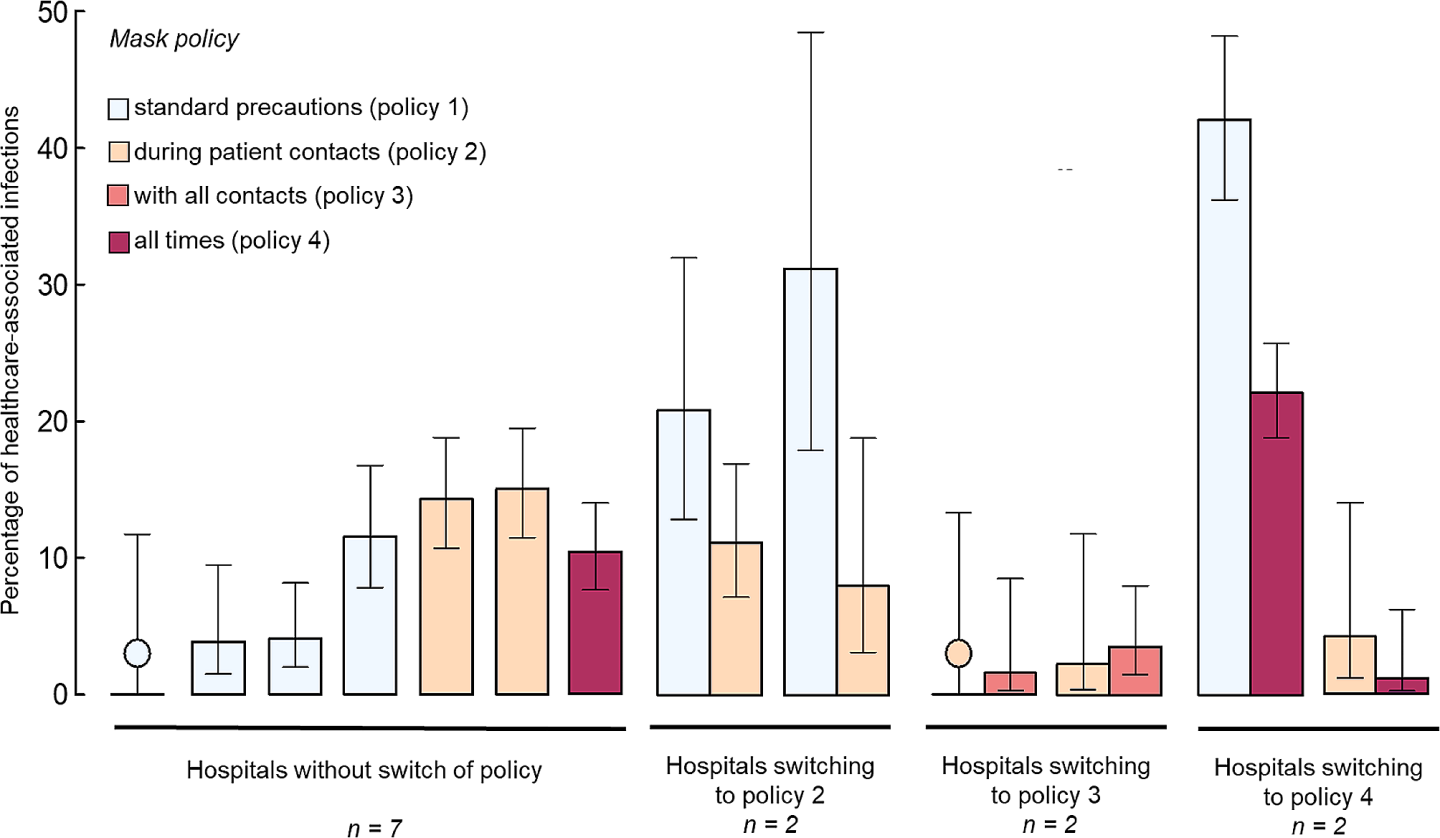
Percentage of healthcare-associated infections by hospital and mask policy, with 95% Wilson CI). Institutions are grouped by the policies switched to. If there were no healthcare-associated infections, a coloured dot indicates the mask policy

For institutions with policy switch, Fig. 4 shows that in 4/6 (66%) institutions the percentage of healthcare-associated SARS-CoV-2 infection decreased with change to a more stringent mask policy. All hospitals switching from policy 1 to a policy requiring at least general masking with patients reported much lower percentages of healthcare-associated SARS-CoV-2 infection after the policy change (RR 0.39, 95% CI 0.30 to 0.49). Conversely, among institutions without change in mask policy, there was no clear association between percentage of healthcare-associated SARS-CoV-2 infection and mask policy (Fig. 4).

## Discussion

In the present investigation, we analysed data for healthcare-associated SARS-CoV-2 infection during the BA.4/5 wave in summer 2022 in dependence of mask and other policies in 13 Swiss hospitals. We could demonstrate the tendency for a decline in healthcare-associated SARS-CoV-2 infections with more stringent mask policies when the percentage of healthcare-associated infection rose. This has been found to correlate with the level of community transmission, which is reflected in our results given the setting of the BA.4/5 wave (14). Although no clear association could be found for the mask policies themselves in the inter-institutional comparison, an intra-institutional switch was associated with a reduction in healthcare-associated SARS-CoV-2 infections. In institutions without a policy switch, the distribution of healthcare-associated infections percentages was similar to community-acquired infections that mirrored the epidemiological curve of the BA.4/5 wave in Switzerland. The course of percentages of healthcare-associated infections in these showed a much slighter increase and even periods of decrease. The factors contributing to this development should be investigated further, but cannot be answered by the data at hand.

We further observed that for other prevention measure policies regarding visitors, HCWs and patients such as repeated testing, social distancing or isolation precautions the majority of institutions were in concordance, with some taking to a more cautious approach. Also, in contrast to mask policies, only few switches were observed regarding other policies.

Overall, our findings suggest that a switch to a more stringent mask policy in acute care institutions is associated with a reduction of healthcare-associated acquisition of SARS-CoV-2. The association of stringent mask policies with prevention of healthcare-associated transmission has been demonstrated for SARS-CoV-2 (15, 16), as well as other respiratory viral diseases, including RSV (17, 18), SARS (19, 20) and influenza (21). For the latter, a strict mask policy for staff on wards with at least three cases led to a 50% reduction in healthcare-associated influenza cases over three consecutive influenza seasons. The acquisition of viral respiratory disease by HCW has been shown to be reduced after introducing the use of masks, not only for SARS-CoV-2 (22–24) but also for Middle East Respiratory Syndrome-Coronavirus (25) and influenza (26). Due to missing data, we could not evaluate the latter relation in the current study.

However, considering the small number of hospitals included in this analysis and given the varying percentages of healthcare-associated infections overall, the association observed should be interpreted with caution. Since a change in mask policy is most commonly part of an intervention bundle, the correlation may not be attributable to mask use exclusively. Implementation of infection prevention bundles may also be a precautionary reaction to a rise in community transmission levels before this translates to an increase of healthcare-associated infections or result from intra-institutional outbreaks. Other factors such as testing and screening policies for patients and HCW, isolation precautions and visitor regulations, room occupancies, vaccination status of both HCW and patients or institutional custom regarding working with confirmed SARS-CoV-2 infections have not been taken into account but must be expected to also influence the rate of healthcare-associated SARS-CoV-2 infections (27–30). Finally, the change in policy creates awareness by influencing the daily routine of HCW, which may also contribute to the association found.

### Limitations

Our study has several limitations. First, these data are from a limited number of institutions in a single country during the 2022 SARS-CoV-2 Omicron Summer wave. They cannot necessarily be generalized to other geographical regions, other viral variants and other epidemiological and seasonal circumstances. Second, due to sample size limitations, we were not able to include other policy measures or vaccination status in our statistical models and other factors, such as regional differences in community transmission levels may influence the frequency of healthcare-associated SARS-CoV-2 infections but could not be taken into account. Third, the reasons for a policy switch were not documented and may also reflect precaution in light of rising community transmission levels or be the reaction to intra-institutional outbreak situations which may confound the results. However, we would expect this to result in a more conservative estimate of healthcare-associated SARS-CoV-2 infections’ percentages and the consistency of our approach ensures the internal validity of our results.

## Conclusion

Our findings support the use of masks in the prevention of healthcare-associated SARS-CoV-2 infection and a switch to a more stringent policy may be beneficial, especially when the rate of healthcare-associated infections increases.

### Electronic Supplementary Material

Below is the link to the electronic supplementary material

Supplementary Material 1

Supplementary Material 2

## Data Availability

The dataset supporting the conclusions of this article are available on request from the Hospital-based surveillance of COVID-19 in Switzerland (CH-SUR) main investigators which can be contacted here [https://www.unige.ch/medecine/hospital-covid/].

## Abbreviations

SARS-CoV-2: severe acute respiratory syndrome coronavirus type 2
COVID-19: coronavirus disease 2019
HCW: healthcare workers
WHO: World Health Organization
CH-SUR: COVID-19 Hospital Based Surveillance System (CH-SUR)
CCER: Geneva Ethics Committee
BASEC: Business Administration System for Ethics Committees
RR: Rate ratio
