# Supplementary material for: Association of institutional masking policies with healthcare-associated SARS-CoV-2 infections in Swiss acute care hospitals during the BA.4/5 wave (CH-SUR study): a retrospective observational study
Source: Antimicrob Resist Infect Control. 2024 Jun 18;13:64. doi: 10.1186/s13756-024-01422-4 (PMC11184728; doi:10.1186/s13756-024-01422-4)
Supplement: Supplementary file 1 — Supplementary Material 1 [file 13756_2024_1422_MOESM1_ESM.docx]

**Supplement 1: Data collection and procession in the CH-SUR database**

The CH-SUR database was designed to capture most of the severe COVID-19 cases in Switzerland by including hospitalized patients from 20 large adult and pediatric hospitals. Laboratory confirmed COVID-19 episodes (including polymerase chain reaction tests and antigen tests) with hospitalization for >24 hours were included. Data were collected by dedicated, centre-located and physician-supervised study personnel and case report forms were stored anonymously in a secure REDCap database. The case report form contained a compulsory part, documenting inclusion criteria, demographics, episode declaration (classification, date of detection and date of symptoms, laboratory sample information), and admission details (hospitalization ward, place of referral, severity at admission, and follow-up forms as well as an optional part, in which the patient’s course of hospitalization could be detailed. To allow for timely surveillance in the pandemic, basic epidemiological information had to be reported within 48 hours after identification, missing information could be added later.

Multiple episodes could be linked to one patient. In case of multiple episodes for one individual, a new episode was defined as readmission >30 days after previous discharge. If multiple case report forms were entered for one episode (i.e. in case of transferal between hospitals), the data were also linked and classified as one infection.

The data were processed through a set of Python (version 3.8.4) and R (version 4.2.0) scripts, which assessed their quality, cleaned and created episode-level data by linking records belonging to the same infection of the same patient.
