# Supplementary material for: Association of institutional masking policies with healthcare-associated SARS-CoV-2 infections in Swiss acute care hospitals during the BA.4/5 wave (CH-SUR study): a retrospective observational study
Source: Antimicrob Resist Infect Control. 2024 Jun 18;13:64. doi: 10.1186/s13756-024-01422-4 (PMC11184728; doi:10.1186/s13756-024-01422-4)
Supplement: Supplementary file 2 — Supplementary Material 2 [file 13756_2024_1422_MOESM2_ESM.docx]

**Supplement 2: Other institutional policies**

Overall, 12 of the 13 included institutions provided additional information on policies regarding testing of HCW and patients, extent and duration of isolation measures and obligations or restrictions for visitors.

***Institutional policies for visitors***

9 institutions (75%) did not enforce restrictions for visitors regarding number or obligatory mask use, 3 of these (25%) switched to a mask obligation during the time investigated, this policy being implemented for 1 other institution (8%). Restrictions regarding the number of daily visitors were enforced by 2 (17%) institutions, 1 (8%) requiring a mask as well. 1 institution did not provide information on visitor policies.

***Institutional policies during the BA.4/5 wave for HCW***

Testing and working policy for HCW remained constant between June 1 and August 31 in all institutions, the majority of 75% (n = 9) testing in case of suspected infection. 2 (15%) institutions performed no testing in symptomatic HCW unless they belonged to a risk group according to FOPH criteria. 1 (8%) institution performed routine screening on wards with high-risk patients.

While 4 (31%) institutions required absence of fever only for HCW to go back to work, 9 institutions (69%) defined a time frame after disease onset in which HCW were not allowed to return to work. The number of days required were 2 (n = 5), 3 (n = 2) and 5 (n =2).

***Institutional policies for patients***

Testing and isolation policies for patients remained constant between June 1 and August 31 in all institutions, all performing tests in case of suspected SARS-CoV-2 infection as well as screenings in asymptomatic patients. In 5 (42%) institutions the trigger of asymptomatic testing was nosocomial exposure, another 4 (33%) tested all patients on admission additionally and the remaining 3 (25%) even performed repeated screening during the hospitalization.

Patients with confirmed infection were isolated for a fixed number of days and had to be free of fever and/or respiratory symptoms in all institutions, 3 (25%) institutions further required a negative test for specific patient groups (e.g. immunocompromised) to revoke isolation. Extent of isolation precautions also varied between institutions. 1 (8%) required droplet precautions only, the 11 had contact precautions in place, 4 (33%) of which required respirator masks and 6 (50%) recommended the use of surgical masks. 1 institution did not give information on isolation practice.


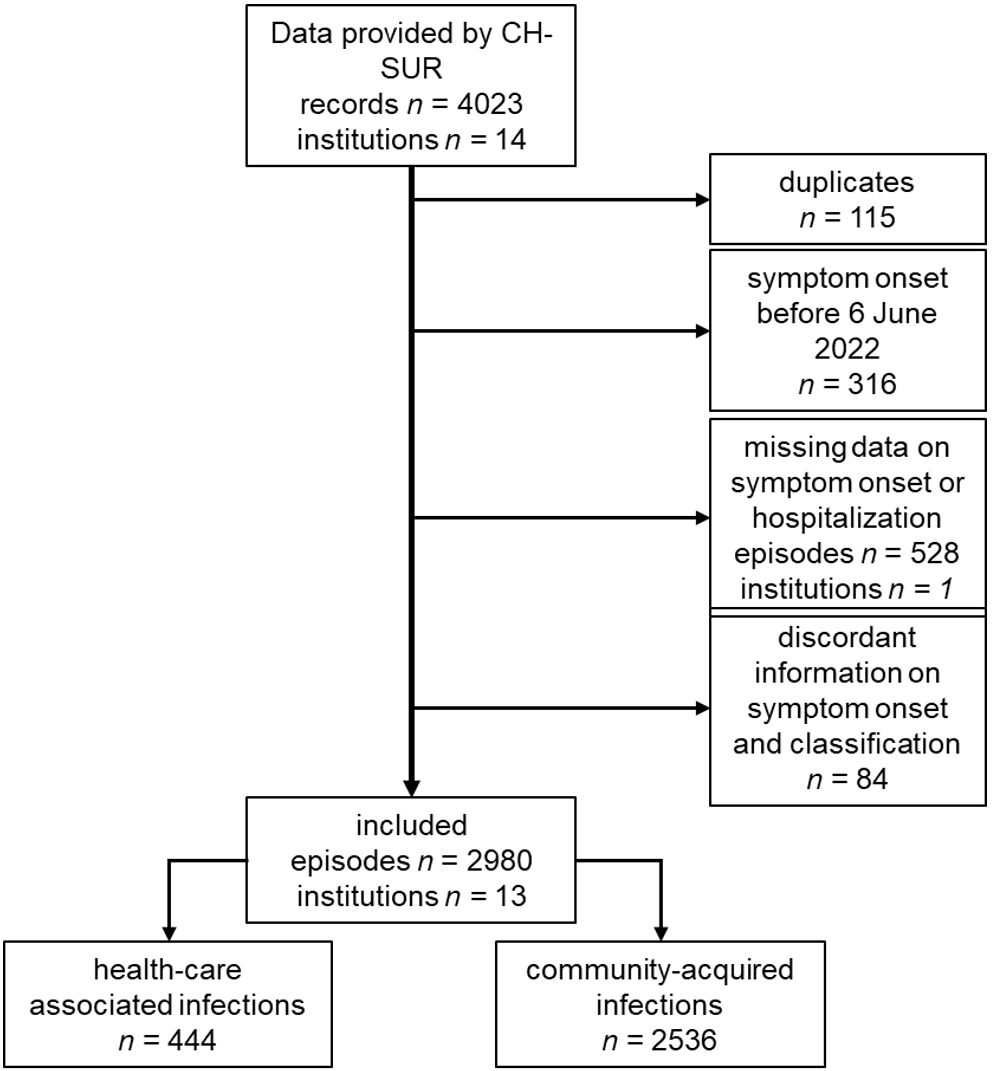


**Figure S1** Flow chart showing the inclusion and exclusion of SARS-CoV-2 episodes retrieved from the CH-SUR database.
